# Advanced Non-Small-Cell Lung Cancer in Elderly Patients: Patient Features and Therapeutic Management

**DOI:** 10.1155/2018/8202971

**Published:** 2018-04-30

**Authors:** Nakano Takayuki, Tanimura Keiko, Uchino Junji, Kaneko Yoshiko, Tamiya Nobuyo, Yamada Tadaaki, Takayama Koichi

**Affiliations:** Department of Pulmonary Medicine, Kyoto Prefectural University of Medicine, 465 Kajii-cho, Kawaramachi-Hirokoji, Kamigyo-ku, Kyoto 602-8566, Japan

## Abstract

Lung cancer has the highest mortality rate among all cancers in most developed countries. The number of elderly patients with lung cancer has been increasing, reflecting the global increase in aging population. Therefore, standard chemotherapeutic regimens for elderly patients with lung cancer need to be established. However, the effectiveness of chemotherapy in elderly patients with advanced non-small-cell lung cancer remains controversial because they are often excluded from clinical trials. Some clinical trials have shown that the therapeutic benefit of a third-generation anticancer drug alone was superior to best supportive care. In contrast, platinum-doublet was superior only in terms of overall survival and progression-free survival, and other trials reported an increased rate of treatment-related death in the elderly patients. In recent years, some novel treatment modalities for lung cancer have been developed and shown to significantly improve the therapeutic outcomes, including targeted therapy for lung cancer harboring driver mutation, combination therapy of angiogenesis inhibitor and cytotoxic agents, and immune checkpoint inhibitor. Although several clinical trials with these agents have shown favorable outcome regardless of age, their safety in the elderly patients has not been established. Herein, we discuss the current clinical status and future prospects in elderly patients with lung cancer.

## 1. Introduction

Lung cancer is among the malignancies with poor prognosis. In 2015, lung cancer was the fifth leading cause of mortality, with the World Health Organization (WHO) reporting 1.7 million deaths worldwide [1]. This mortality rate was the highest among all cancers of the organs. Given that old age increases the risk for developing lung cancer, the proportion of elderly patients with lung cancer has also been increasing, reflecting the global increase in aging population [2]. Such trend is more prominent in Japan because of its high elderly population, and 75% of patients who died of lung cancer are the elderly aged 70 years or older [3]. Although elderly patients with lung cancer can also be treated with standard therapy, only few clinical trials target elderly patients. Thus, the therapeutic management for lung cancer has not been properly assessed for this patient group. Some clinical trials showed an increase in the incidence of adverse events and poor efficacy of standard treatment modalities; by contrast, other drugs were reported to achieve favorable antitumor effects in both elderly and young patients. However, most clinical trials that reported these promising studies excluded elderly patients with poor physical state [4].

In recent years, drug therapy for advanced lung cancer has rapidly progressed. Due to the relatively mild adverse events compared to conventional cytotoxic agents, drug therapy, such as molecular targeted drugs and immune checkpoint inhibitors, has been widely considered to be appropriate for elderly patients with lung cancer for whom therapy is indicated via biomarker testing [5]. In this study, we discuss the current state of and the issues to be addressed in drug therapy for elderly patients with advanced non-small-cell lung cancer (NSCLC).

## 2. Features of Elderly Patients with Lung Cancer

Compared with young patients with cancer, the elderly has several characteristics that need to be considered during treatment, including reduced ability for performance of activities of daily living, history of multiple comorbid diseases, decline in organ function, reduction in cognitive function, and changes in social environment. In lung cancer, cigarette smoking (which is the leading cause of lung cancer) and chronic obstructive pulmonary disease (COPD, which is the most common comorbidity of lung cancer) often limit air flow, decreasing the ability for physical activity. The adverse effects of cigarette smoke accumulates, and the risk for COPD increases with age [6]. Moreover, elderly patients with lung cancer tend to have cardiac comorbidities such as congestive heart failure, which can be a problem in chemotherapy, particularly for chemotherapeutics that need to be administered with high-volume hydration [7]. Furthermore, Repetto et al. reported that the risk of cognitive disorders in patients with advanced lung cancer increases with age. The percentage of patients with low mini-mental state examination score is at 29.0% among those aged 74 years or younger versus 78.4% among those aged over 85 years [8].

These factors should be carefully considered in developing a chemotherapy plan for elderly patients with lung cancer.

## 3. Changes in Physiological Function and Pharmacokinetics in the Elderly

Organ and physiological functions decrease over time after the age of 40 years. Because drugs are excreted from the kidney or liver, pharmacokinetics are affected by decreases in renal or hepatic bioactivity.

Renal function is easily affected by decreased renal blood flow and arteriosclerotic change due to aging, and the rate of creatinine clearance becomes two times slower after the age of 45 years [9]. Therefore, proper dosage adjustment is required during drug administration in elderly patients.

Multiple factors, such as hepatic blood flow, metabolizing enzyme activity, and ratio of an unbound drug with plasma protein, affect the pharmacokinetics of drugs excreted mainly through the hepatic/biliary metabolism. Physiological changes in hepatic function, reductions in hepatic blood flow caused by reduced cardiac output, and decreases in the metabolizing enzyme CYP due to aging have been reported [10]. In addition, renal dysfunction significantly affects the functions of drug-metabolizing enzymes and transporters in the liver [11]. Given that elderly patients commonly develop multiple types of organ dysfunction, a reduced capacity for drug clearance can cause persistently high drug levels in the blood, which may lead to an increase in drug toxicity. Therefore, appropriate dosage adjustment and careful monitoring are required in this patient population.

## 4. Chemotherapy for Elderly Patients with Advanced Non-Small-Cell Lung Cancer

### 4.1. Cytotoxic Chemotherapy

In the 20th century, evidence on the efficacy of chemotherapy for advanced NSCLC in elderly patients was limited. However, the results of two phase III trials, namely, the Elderly Lung Cancer Vinorelbine Italian Study and the Multicenter Italian Lung Cancer in the Elderly Study (MILES), showed that the therapeutic benefits of a third-generation anticancer drug alone, such as vinorelbine (VNR) and gemcitabine (GEM), are superior to best supportive care alone [12, 13]. In the phase III WJTOG9904 trial conducted in Japan, although no significant difference in outcomes was obtained, docetaxel (DTX) alone extended overall survival (OS) and progression-free survival (PFS) compared to VNR alone. Therefore, DTX has been recommended in Japan's guidelines for treatment of lung cancer in the elderly [14]. However, the most superior third-generation anticancer drug remains undetermined. After 2010, subgroup analysis of pemetrexed and nanoparticle albumin-bound paclitaxel demonstrated favorable results in elderly groups. Phase III trials are now underway to confirm their therapeutic benefit in nonsquamous cell carcinoma [19, 20] (Table 1).

The application of combined therapy with a platinum agent is controversial. A meta-analysis and subgroup analysis of the Cochrane Database of Systematic Review on 51 randomized controlled trials showed that chemotherapeutics combined with a platinum agent extended OS compared to that of a nonplatinum agent; however, the toxicity also tended to worsen [42]. The results of the IFCT-501 phase III trial, in which combination therapy of carboplatin (CBDCA) plus PTX was compared with VNR or GEM monotherapy, showed that CBDCA plus PTX was superior in terms of OS and PFS, but the rate of treatment-related death also tended to be high (4.4%) [15]. Therefore, the recommended therapeutic regimen for elderly population varies per country: monotherapy is recommended in Japan, while combined therapy with CBDCA is recommended in Western countries for patients who are in good general condition. Meanwhile, pooled analysis of two phase III trials, namely, MILES-3 and MILES-4, showed that combination treatment with cisplatin (CDDP) resulted in a more favorable response rate compared to monotherapy using a third-generation anticancer drug in patients with advanced lung cancer with a performance status (PS) of 0-1 and aged 70 years or older. Such results have been presented in the 2017 American Society of Clinical Oncology convention. PFS was extended in the group treated with combination CDDP, but no significant difference was observed in OS. In addition, the rates of toxicity, febrile neutropenia, and body malaise were also higher in the group treated with combination CDDP therapy [16]. The pharmacokinetics of CDDP is similar between the elderly and young patients [43]. Although evidence for the active recommendation of CDDP administration in elderly patients is lacking, CDDP should still be considered for such patients given that the drug has been shown to yield beneficial results.

### 4.2. EGFR Tyrosine Kinase Inhibitors

Active mutation in the* epidermal growth factor receptor (EGFR)* gene is highly correlated with the efficacy of EGFR tyrosine kinase inhibitors (EGFR-TKI).* EGFR* is among the indispensable biomarkers in selecting the therapeutic modality for NSCLC patients. Several phase III comparative studies, including NEJ002, have been conducted and have shown that EGFR-TKI is more effective than conventional cytotoxic agents as first-line therapy for* EGFR* mutation-positive lung cancer [44]. Although the median age of the subjects in these trials was approximately 60 years, several elderly patients were included in these studies, and subset analysis of PFS showed a trend in favor of EGFR-TKI in the elderly group [21–23].

By contrast, although only few comparative studies of EGFR-TKI targeting only elderly patients with* EGFR* mutation-positive lung cancer have been conducted, the results of the NEJ001 trial, which included patients with a PS of 3 or 4 and aged younger than 74 years, those with a PS of 2–4 and aged between 75 and 79 years, and those with a PS of 1–4 and aged older than 80 years, showed favorable outcome improvement in PS in most patients in the group given gefitinib (PFS: 6.5 months; OS: 17.8 months) [45]. In addition, the results of the NEJ003 trial that targeted patients with a PS of 0–2 and older than 75 years showed a response rate of 74%, a median PFS of 12.3 months, a one-year survival rate of 83.9%, and a two-year survival rate of 58.1% in the group administered with gefitinib [46]. The results of a pooled analysis combining the NEJ001, NEJ002, and NEJ003 trials that limited the subjects to elderly patients older than 70 years showed that PFS was longer in the gefitinib administration group than in the chemotherapy group (CBDCA plus PTX), but no significant difference was observed for OS [47]. These results are similar to those of subgroup analyses of elderly patients in phase III comparative studies and indicate the high efficacy of EGFR-TKI as treatment for* EGFR* mutation-positive lung cancer, even in elderly patients with poor PS. Therefore, EGFR-TKI has been highly recommended and widely used in elderly patients both in Japan and in Western countries.

However, most trials that included only elderly patients used gefitinib, and studies using erlotinib or second- or third-generation EGFR-TKI are limited. A comparative analysis between patients aged older and younger than 75 years showed that the efficacy of erlotinib is similar in the elderly group and in the younger group, and the adverse effects were manageable [48]. Meanwhile, although no comparative studies regarding the second-generation EGFR-TKI afatinib and dacomitinib and the third-generation EGFR-TKI osimertinib have been performed specifically for elderly patients, the results of subset analyses have suggested the efficacy of such drugs in elderly patients [24–28]. In particular, osimertinib, which is effective against the* EGFR-T790M* mutation that accounts for nearly 50% of susceptibility to EGFR-TKI, is known for its efficacy as well as high tolerability, and studies on its applicability in elderly patients are expected (Table 2).

### 4.3. *ALK* Inhibitors

*Anaplastic lymphoma kinase (ALK) *inhibitors, such as crizotinib, alectinib, and ceritinib, are currently developed and are among the treatment options for* ALK* rearrangement-positive lung cancer, which accounts for 3%–5% of all cases of NSCLC. However, at present, no comparative study for such drug that targets only elderly patients has been performed. In a subset analysis of the PROFILE 1014 trial, 55 of the 343 patients were elderly (older than 65 years); the results of this analysis showed the efficacy of crizotinib and a prolonged PFS in the elderly group [29]. In addition, the results of a subgroup analysis of the ALEX study, which compared the efficacy between crizotinib and alectinib as first-line therapy, showed that the PFS was extended in both patients aged younger than 75 years and older than 75 years, and the extension was higher in the alectinib group compared to those in the crizotinib group. An analysis of the J-ALEX study that targeted the Japanese population also showed similar results [30, 31].

### 4.4. Angiogenesis Inhibitors

Combining angiogenesis inhibitors with cytotoxic drugs or molecular targeted drugs yields additive effects, which tends to be different in elderly patients from young patients. A subset analysis of the AVAiL phase III trial of bevacizumab showed the efficacy of combination of cytotoxic agents with bevacizumab in patients aged older than 65 years [32]. Meanwhile, a subset analysis of the ECOG4599 trial and pooled analysis of the PointBreak trial showed no additive effect from the combination of cytotoxic agents with bevacizumab in elderly patients aged older than 70 years; however, the toxicity tended to increase [33]. In the SAiL trial, which is an observational study in Europe, the efficacy and safety of combining angiogenesis inhibitors with cytotoxic drugs or molecular targeted drugs in patients aged 70 years or older were similar to those in young patients [34]. The results of the ARIES trial, which is an observational study in the US, showed that neither the incidence of adverse events nor PFS in patients older than 75 years was different from those of young patients; however, OS was shorter in the elderly patients [35].

Concerning ramucirumab (RAM), which is an anti-vascular endothelial growth factor receptor-2 antibody, the results of the REVEL phase III trial showed that OS, PFS, and response rate in the DTX plus RAM group were superior to the DTX alone group. In a subgroup analysis of the REVEL trial, the additive effect of RAM to DTX was not observed in elderly patients, and the incidence of adverse events higher than grade 3 tended to be higher in the DTX plus RAM group [36, 37] (Table 3).

Evidence for recommending anti-VEGF therapy for elderly patients with NSCLC is limited. Although anti-VEGF antibodies yield significant additive effects in conditions where VEGF is a key factor, such as metastasis to the central nervous system [49, 50], malignant pleural effusion [51], and pericardial effusion, its use still requires caution.

### 4.5. Immune Checkpoint Inhibitors

Immune checkpoint inhibitors, including nivolumab, which was approved in 2015, show favorable outcomes in advanced lung cancer. Although the incidence of immune-related adverse events is higher in immune checkpoint inhibitors than conventional cytotoxic agents, the incidence of adverse events that lead to deterioration in general condition, such as anorexia, malaise, and myelosuppression, is low. Therefore, immune checkpoint inhibitors are considered safe for elderly patients. However, no clinical trial regarding the use of immune checkpoint inhibitors exclusive to elderly patients has been performed. The results of a subset analysis of the CheckMate 017 study showed that, in the group of patients aged 65–74 years, improvement in the survival rate was almost similar to that in patients younger than 65 years. Meanwhile, no efficacy was observed in patients 75 years or older. However, given the inclusion of a small number of patients in the elderly group, which affected the results, the efficacy of the treatment cannot be concluded to be inferior based on the analysis results [38, 39]. In addition, pembrolizumab, an anti-programmed cell death-1 (PD-1) antibody, was shown to be superior to combined therapy with a platinum agent as first-line treatment for diseases with more than 50% programmed cell death ligand 1 (PD-L1) expression in a tumor surface. A subgroup analysis regarding PFS showed favorable results from immune checkpoint inhibitors independent of age in patients older than 65 years and those who were younger [40]. Meanwhile, the results of a subgroup analysis in the KEYNOTE-010 study, which is a comparative trial between pembrolizumab and DTX as second-line therapy, did not show significant improvement in the OS of patients aged 65 years or older (Table 4) [41].

Studies have shown that, compared to young people, alterations in the number of T cells in the elderly are minimal in immunosenescence due to aging but acquired antigen-specific immune function declines. In particular, studies have reported hastened immune aging, particularly in patients with cancer [52–54]. However, some elderly patients respond favorably to immune checkpoint inhibitors despite their age. Therefore, the mechanism by which the immune response to tumors, which is activated by immune checkpoint inhibitors, is affected by aging remains unclear, and further studies are necessary.

## 5. Conclusion

With an increase in the number of elderly patients with lung cancer, comprehensive assessments of problems specific to elderly patients and provision of adequate and appropriate therapy are rising concerns. A number of clinical trials targeting elderly patients are currently underway, and further evidence is anticipated.

## Figures and Tables

**Table 1:** Clinical trials of cytocidal anticancer drugs for elderly patients with NSCLC.

Study, author	*n*.	Treatment	RR (%)	OS (month)	PFS (month)	Neutropenia G3-4 (%)
*Phase III trials*						
ELVIS [12]	78	VNR	19.7	6.5	NR	NR
	96	BSC	-	4.9	-	-
MILES [13]	232	VNR + GEM	21	7.6	4.8	18
	233	VNR	18	8.8	4.5	25
	233	GEM	16	6.6	4.3	8
WJTOG9904 [14]	88	DTX	22.7	14.3	5.5	82.9
	91	VNR	9.9	9.9	3.1	69.3
IFCT-0501 [15]	225	CBDCA + PTX	27.1	10.3	6	48.4
	226	VNR or GEM	10.2	6.2	2.8	12.4
MILES-3/MILES-4 [16]	263	CDDP + GEM or PEM	15.5	9.6	4.6	Significantly higher and more sever in CDDP group.
	268	GEM or PEM	8.5	7.5	3	
JCOG0803 [17]	139	CDDP + DTX	34.4	14.8	4.7	10.1
	137	DTX	24.6	13.3	4.4	88.8
JCOG0207 [18]	63	CDDP + DTX	55	17	6.2	14.3
	63	DTX	26.2	10.7	3.7	4.8
*Subset analysis of elderly group*						
Socinski et al. [19]	73	nab-PTX + CBDCA	34	8	19.9	55
	81	PTX + CBDCA	24	6.8	10.4	73
PARAMOUNT [20]	52	PEM	42	13.7	6.4	17
	40	Placebo	43	12.1	3	-

RR = response rate, OS = overall survival, PFS = progression free survival, NR = not reported, VNR = vinorelbine, BSC = best supportive care, GEM = gemcitabine, DTX = docetaxel, PTX = paclitaxel, CDDP = cisplatin, CBDCA = carboplatine, nab-PTX = nanoparticle albumin-bound paclitaxel, and PEM = pemetrexed.

**Table 2:** Subset analysis of elderly population in clinical trials of EGFR-TKIs or ALK inhibitors for NSCLC.

Study	Treatment	Age	*n*.	HR for OS (95% CI)	HR for PFS (95% CI)
*(A) EGFR-TKIs*					
IPASS [21]	Gefitinib	≥65	NR	NR	0.58 (0.45–0.76)
		<65	NR	NR	0.81 (0.70–0.95)
OPTIMAL [22]	Erlotinib	≥65	38	NR	0.17 (0.07–0.43)
		<65	116	NR	0.19 (0.11–0.31)
EUROTAC [23]	Erlotinib	≥65	88	NR	0.28 (0.16–0.51)
		<65	85	NR	0.44 (0.25–0.75)
LUX-Lung 3 [24, 25]	Afatinib	≥65	134	0.73 (0.43–1.21)	0.64 (0.39–1.03)
		<65	211	0.82 (0.57–1.19)	0.53 (0.36–0.76)
LUX-Lung 6 [25, 26]	Afatinib	≥65	86	0.60 (0.33–1.10)	0.16 (0.07–0.40)
		<65	278	0.87 (0.64–1.20)	0.30 (0.21–0.43)
ARCHER1050 [27]	Dacomitinib	≥65	94	NR	0.69 (0.48–0.99)
		<65	133	NR	0.51 (0.39–0.69)
AURA3 [28]	Osimertinib	≥65	177	NR	0.34 (0.23–0.50)
		<65	242	NR	0.38 (0.28–0.54)
*(B) ALK inhibitors*					
PROFILE1014 [29]	Crizotinib	≥65	55	NR	0.90 (0.43–1.87)
		<65	288	NR	0.45 (0.29–0.70)
ALEX [30]	Alectinib	≥65	70	NR	0.45 (0.24–0.87)
		<65	233	NR	0.48 (0.34–0.70)
J-ALEX [31]	Alectinib	≥75	22	NR	0.28 (0.06–1.19)
		<75	285	NR	0.34 (0.21–0.56)

HR = hazard ratio, OS = overall survival, PFS = progression free survival, and NR = not reported.

**Table 3:** Subset analysis of elderly population in clinical trials of angiogenesis inhibitors for NSCLC.

Study	Age	*n*.	Treatment	OS (month)	HR for OS (95% CI)	PFS (month)	HR for PFS (95% CI)
AVAiL [32]	≥65	103	CG + BEV (15 mg/kg)	NR	0.88	NR	0.84
	<65	248	CG + BEV (15 mg/kg)	NR	1.09	NR	0.85
ECOG4599, PointBreak [33]	≥75	114	BEV + CP	9.6	1.10 (0.74–1.60)	5.6	0.95 (0.62–1.44)
	<75	787	BEV + CP	13.4	0.76 (0.66–0.87)	6.1	0.69 (0.60–79)
SAiL [34]	>65	623	Chemotherapy + BEV	14.6	NR	8.2	NR
	≤65	1589	Chemotherapy + BEV	14.6	NR	7.6	NR
ARIES [35]	≥65	1013	Chemotherapy + BEV	12.1	1.17 (1.06–1.28)	6.8	1.01 (0.92–1.10)
	<65	954	Chemotherapy + BEV	14.2	1	6.4	1
REVEL [36, 37]	≥70	127	RAM + DTX	NR	1.07 (0.80–1.43)	NR	0.94 (0.73–1.22)
	<70	591	RAM + DTX	NR	0.81 (0.70–0.94)	NR	0.73 (0.64–0.83)

RR = response rate, OS = overall survival, PFS = progression free survival, HR = hazard ratio, NR = not reported, CG = carboplatin/gemcitabine, BEV = bevacizumab, CP = carboplatin/paclitaxel, DTX = docetaxel, and RAM = ramucirumab.

**Table 4:** Subset analysis of elderly population in clinical trials of immune checkpoint inhibitors for NSCLC.

Study	Age	*n*.	HR for OS (95% CI)	HR for PFS (95% CI)
*Nivolumab*				
CheckMate017 [38]	≥75	29	1.85 (0.76–4.51)	1.76 (0.77–4.05)
	65–74	91	0.56 (0.34–0.91)	0.51 (0.32–0.82)
	<65	152	0.52 (0.35–0.75)	0.62 (0.44–0.89)
CheckMate057 [39]	≥75	43	0.97 (0.49–1.95)	0.90 (0.43–1.87)
	65–74	200	0.94 (0.69–1.27)	0.63 (0.45–0.89)
	<65	339	0.89 (0.70–1.13)	0.81 (0.62–1.04)
*Pembrolizumab*				
KEYNOTE024 [40]	≥65	164	NR	0.90 (0.43–1.87)
	<65	141	NR	0.45 (0.29–0.70)
KEYNOTE010 [41]	≥65	429	0.76 (0.57–1.02)	NR
	<65	604	0.63 (0.50–0.79)	NR

RR = response rate, OS = overall survival, PFS = progression free survival, and NR = not reported.

## Data Availability

All date related to this paper are available in PubMed.
